# Quantitative lung SPECT applied on simulated early COPD and humans with advanced COPD

**DOI:** 10.1186/2191-219X-3-28

**Published:** 2013-04-19

**Authors:** Pernilla Norberg, Hans Lennart Persson, Gudrun Alm Carlsson, Björn Bake, Magnus Kentson, Michael Sandborg, Agnetha Gustafsson

**Affiliations:** 1 Department of Medical and Health Sciences, Linköping University, and Center for Medical Image Science and Visualization, and Medical Radiation Physics, County Council of Östergötland, Linköping, SE-581 85, Sweden; 2 Department of Medical and Health Sciences, Linköping University, and Department of Respiratory Medicine, County Council of Östergötland, SE-581 85, Linköping, Sweden; 3 Department of Medical and Health Sciences, Linköping University, and Center for Medical Image Science and Visualization, Linköping, SE-581 85, Sweden; 4 Department of Internal Medicine, Institute of Medicine, Sahlgrenska Academy at University of Gothenburg, Göteborg, SE-413 46, Sweden; 5 Division of Pulmonary Medicine, Ryhov Hospital, SE-551 85, Jönköping, Sweden; 6 Department of Medical and Health Sciences, Linköping University, and Center for Medical Image Science and Visualization, and Department of Clinical Physiology, County Council of Östergötland, SE-581 85, Linköping, Sweden

**Keywords:** Quantitative lung SPECT, Ventilation, Iterative reconstruction, Lung disorder, Monte Carlo, COPD

## Abstract

**Background:**

Reduced ventilation in lung regions affected by chronic obstructive pulmonary disease (COPD), reflected as inhomogeneities in the single-photon emission computed tomography (SPECT) lung image, is correlated to disease advancement. An analysis method for measuring these inhomogeneities is proposed in this work. The first aim was to develop a quantitative analysis method that could discriminate between Monte Carlo simulated normal and COPD lung SPECT images. A second aim was to evaluate the ability of the present method to discriminate between human subjects with advanced COPD and healthy volunteers.

**Methods:**

In the simulated COPD study, different activity distributions in the lungs were created to mimic the healthy lung (normal) and different levels of COPD. Gamma camera projections were Monte Carlo simulated, representing clinically acquired projections of a patient who had inhaled 125 MBq ^99m^Tc-Technegas followed by a 10-min SPECT examination. Reconstructions were made with iterative ordered subset expectation maximisation. The coefficient of variance (CV) was calculated for small overlapping volumes covering the 3D reconstructed activity distribution. A CV threshold value (CV_T_) was calculated as the modal value of the CV distribution of the simulated normal. The area under the distribution curve (AUC), for CV values greater than CV_T_, AUC(CV_T_), was then calculated. Moreover, five patients with advanced emphysema and five healthy volunteers inhaled approximately 75 MBq ^99m^Tc-Technegas immediately before the 20-min SPECT acquisition. In the human study, CV_T_ was based on the mean CV distribution of the five healthy volunteers.

**Results:**

A significant difference (*p* < 0.001) was found between the Monte-Carlo simulated normal and COPD lung SPECT examinations. The present method identified a total reduction of ventilation of approximately 5%, not visible to the human eye in the reconstructed image. In humans the same method clearly discriminated between the five healthy volunteers and five patients with advanced COPD (*p* < 0.05).

**Conclusions:**

While our results are promising, the potential of the AUC(CV_T_) method to detect less advanced COPD in patients needs further clinical studies.

## Background

Chronic obstructive pulmonary disease (COPD) is characterised by obstructed airways and parenchymal destruction. Characteristically, varying degrees of abnormalities are found in different parts of the COPD lung, and some parts of the lung may even be normal. Consequently, abnormal ventilation distribution is the first abnormality to be detected in the early stages of the disease [1].

COPD starts with inflammation and obstruction in peripheral airways [2,3] where the resistance is very low [4]. Therefore, conventional lung function tests (spirometry) are insensitive [5]. Imaging techniques primarily aim at localising lesions, but as these techniques become digital, new interpretative possibilities are now arising. By using high-resolution computed tomography (HRCT), mild emphysema may be assessed by quantification of the density distribution [6]. Although computed tomography is an excellent method for identifying anatomical changes in the lung tissue, it provides little information about lung function reduction [6]. In contrast, lung function reduction is both imaged and assessed directly by lung single-photon emission computed tomography (lung SPECT), a method often used in diagnosis of pulmonary embolism [7,8]. Hence, previous reports have described ventilation and perfusion SPECT as a sensitive method of detecting early changes in COPD [9]. Moreover, SPECT findings correlate significantly with emphysema scored by HRCT and lung function tests [9].

It is common to interpret lung SPECT images qualitatively. However, quantitative information obtained from a SPECT examination has the potential to provide much more information. Different quantitative methods for assessment of lung SPECT images have been reported [10-16]. Xu et al. [11] quantified inhomogeneities in ventilation SPECT images of COPD patients using 50 MBq of Technegas (Vita Medical Limited, Sydney, Australia), measuring the coefficient of variance (CV) in the lung elements. In that study, transaxial SPECT slices (1-cm thickness with 3.5-cm spacing) were acquired using a low-energy, general-purpose collimator, filtered back-projection with non-homogeneous attenuation correction and a 2D Hann post-filter. These were then divided into 2 × 2 × 1 cm^3^ elements. By that method, it was possible to separate non-smoking healthy subjects and ‘healthy smokers’ from COPD patients. Importantly, however, that method was not sensitive enough to discriminate between healthy non-smokers and healthy smokers. Our overall aim is to develop a quantitative method sensitive enough to discriminate between these two groups. Therefore, the present study is currently followed by a larger clinical study evaluating the ability of the method used in this paper to differentiate between healthy non-smokers and healthy smokers.

The present study had two aims. Firstly, we wanted to develop a quantitative method using Monte Carlo simulated lung SPECT images of phantom lungs that could discriminate between uniform (healthy, simulated normal) and non-uniform (non-healthy, simulated COPD) activity distributions corresponding to COPD lung changes of varying severity. Secondly, we wished to evaluate the ability of the same method in a clinical environment to differentiate between human subjects with advanced COPD and healthy volunteers.

## Methods

### Lung phantom and Monte Carlo simulations

**Figure 1:**
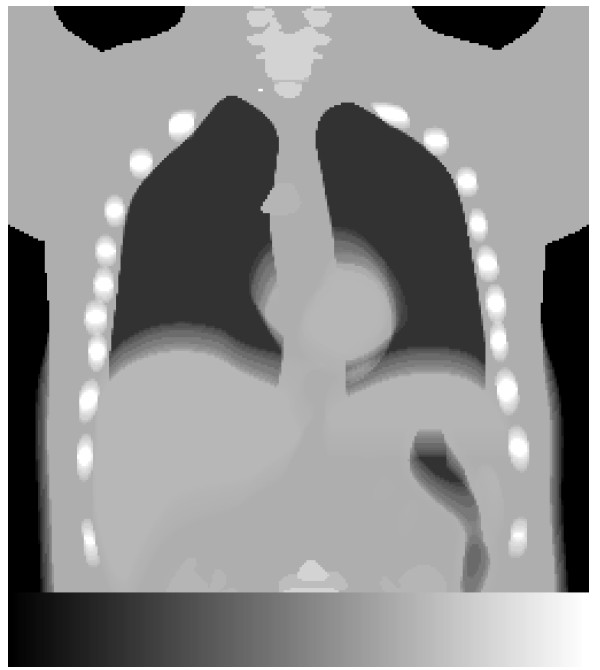
**A cross-section of the phantom visualising selected tissues, respiratory motion and heartbeat.** A coronal slice of the phantom in units of linear attenuation coefficients, together with used grey scale ranging from *μ* = 0 cm^−1^ as black to *μ* = 0.219 cm^−1^ as white.

The NCAT phantom [17] with a lung volume (air, blood and parenchyma) of 4.2 litres was used. The lung volume corresponded to a 65-year-old male (70 kg, 180 cm) in a supine position and in the middle of the respiratory cycle [18,19]. The arms were held over the head (see Figure 1). The respiratory motion and heartbeat of the phantom were employed throughout the study, i.e. the phantom was dynamic. The phantom consisted of a 256 × 256 × 256 matrix with a voxel size of 1.65 × 1.65 × 1.65 mm^3^. Muscle, fat, lung, spine bone, rib bone, blood and heart tissues were selected. The densities and elemental compositions of these tissues were obtained from ICRP 89 [20] and the photon interaction cross sections from Berger et al. [21].

The simulated healthy distribution, in the following called normal, was represented by a homogeneous activity distribution in the phantom lung (the ‘uniform’ distribution). Simulated COPD lung changes of varying severity were represented by eight different non-homogeneous activity distributions. Small spherical lesions were chosen to create inhomogeneous ventilation distributions. These distributions are defined in Table 1. The activity concentrations in the lesions were 50%, 25% or 0% of the healthy surrounding lung tissue. The reduced activity levels in the lesions reflect the magnitude of the reduced ventilation. No activity was located elsewhere in the body. The density of the lesions was the same as for the lung. In Table 1, coronal slices are shown including motion artefacts to illustrate the distributions observed by the gamma camera during projection acquisition.

**Table 1:** Phantom activity distribution notations and their descriptions

	**Distribution notation**	**Coronal slice**	**Number**	**Distribution description**	**Total reduction of ventilation (%)**
Simulated normal	Uniform		1	Homogeneous activity distribution	0
Simulated COPD	1 cm^50%^ 10% evenly		2	Lesions with a diameter of 1 cm with 50% activity concentration, evenly distributed over the lung volume, occupying 10% of the total lung volume.	5
	1 cm^50%^ 12% centred		3	Lesions with a diameter of 1 cm with 50% activity concentration, centred to the large bronchial tubes, occupying 12% of the total lung volume.	6
	1 cm^0%^ 10% evenly		4	Lesions with a diameter of 1 cm with 0% activity concentration, evenly distributed over the lung volume, occupying 10% of the total lung volume.	10
	1 cm^0%^ 12% centred		5	Lesions with a diameter of 1 cm with 0% activity concentration, centred to the large bronchial tubes, occupying 12% of the total lung volume.	12
	2 cm^50%^ 10% evenly		6	Lesions with a diameter of 2 cm with 50% activity concentration, evenly distributed over the lung volume, occupying 10% of the total lung volume.	5
	2 cm^0%^ 10% evenly		7	Lesions with a diameter of 2 cm with 0% activity concentration, evenly distributed over the lung volume, occupying 10% of the total lung volume.	10
	2 cm^50%^ 48% evenly		8	Lesions with a diameter of 2 cm with 50% activity concentration, evenly distributed over the lung volume, occupying 48% of the total lung volume.	24
	2 cm^25%^ 48% evenly		9	Lesions with a diameter of 2 cm with 25% activity concentration, evenly distributed over the lung volume, occupying 48% of the total lung volume.	36

Coronal slices of the phantom include simulated motion artefacts.

SPECT projections from these activity distributions were Monte Carlo simulated using the software SIMIND, version 4.9d [21,22]. The projection data incorporated the effects of non-uniform attenuation, scatter and motion blurring. The isotope ^99m^Tc was used and the energy window was set between 130 and 154 keV. The gamma camera rotation orbit was non-circular, corresponding to the auto-contouring system used in our clinic. The centre of rotation to collimator distance varied between 17 and 25 cm.

Simulations were made for a GE Infinia gamma camera (9.5-mm thick NaI detector) equipped with a low-energy high-resolution collimator (LEHR). Projections were collected at 128 different angles, equally spaced, over 360°, in 128×128 matrices (3.30 × 3.30 mm^2^ per detector element). A total of 1.8 × 10^10^ photons were simulated with the homogeneous activity concentration (the simulated normal) resulting in projections with very low noise levels. The coefficient of variance CV (see Equation 1 in Additional file 1) of one pixel element within the high-count area of the lung in one single projection determined from three consecutive simulations was 0.5%.

### Normalisation and statistical noise

The aim of generating low-noise projections was to be able to adjust the mean number of total counts to that representative of a clinical study, and to generate Poisson noise [23,24] typical of realistic projections. The mean number of counts in the simulated projections was set to 3.635 × 10^6^. This value was based on a virtual administered activity of 125 MBq for each activity distribution, an acquisition time of 10 s per projection and the average sensitivity (cps MBq^−1^) of 20 planar lung perfusion studies at Linkoping University Hospital performed in 2009. The acquisition parameters used then were the following: ^99m^Tc macro-aggregated albumin, LEHR collimator and an energy window of 130-154 keV. Before reconstruction, the mean number of counts in individual pixel elements in the projections was replaced by random deviates drawn from a Poisson distribution.

For each activity distribution, 20 noise realisations were created, imitating 20 SPECT acquisitions of the same activity distribution. The CV value of one pixel element within the high-count area of the lung in one projection, based on the 20 noise realisations of the simulated normal, was about 15%.

### Human subjects and their data acquisition

The patient group consisted of five grade 3 (FEV1/VC < 0.70, 30% ≤ FEV_1_ < 50% of predicted) and 4 (FEV1/VC < 0.70, FEV_1_ < 30% of predicted) [25-27] COPD patients (P1 to P5), registered at the department of Pulmonary Medicine, Linkoping University Hospital, all with advanced emphysema demonstrated on HRCT. Mean values ± 1 SD (expressed as percentage of predicted values) for vital capacity (VC), residual volume (RV), total lung capacity (TLC) and the diffusion capacity of the lung for carbon monoxide (DL_CO_) for the patient group were 84 ± 8, 211 ± 39, 127 ± 9 and 38 ± 7, respectively. The group of healthy volunteers consisted of five (H1 to H5) life-long non-smokers (>40 years old, FEV_1_ >80% predicted), without history of allergy. Obstruction was defined as FEV_1_/VC < 0.7 for ages <65 years and <0.65 for ages ≥65 years according to the Swedish guidelines for COPD [28]. Corresponding mean values ± 1 SD for the volunteer group were 104 ± 11, 126 ± 10, 110 ± 8 and 99 ± 16, respectively. All human subjects were included after informed and written consent were obtained. Additional characteristics of the subjects are presented in Table 2. The regional Ethics Review Board at the University of Linkoping approved the study protocol.

**Table 2:** **Subjects’ gender, age, FEV**_**1**_**, FEV**_**1**_**/VC and AUC(CV**_**T**_**)**

**Subject**	**Label**	**Gender**	**Age (years)**	**FEV**_**1 **_**(% predicted)**	**FEV**_**1**_**/VC**	**AUC(CV**_**T**_**) (%)**
Healthy volunteers	H1	F	51	132	0.85	52
	H2	F	68	98	0.77	75
	H3	M	75	122	0.73	57
	H4	M	69	89	0.69	72
	H5	M	50	111	0.83	71
Patients	P1	M	81	43	0.33	99
	P2	M	84	30	0.25	100
	P3	F	71	22	0.21	100
	P4	F	73	51	0.43	100
	P5	F	52	28	0.30	100

*FEV*_*1*_, forced expiratory volume in one second (% predicted; predicted normal values according to Hedenstrom [26,27]), obstruction was defined as *FEV*_*1*_*/VC* < 0.7 for ages <65 years and <0.65 for ages ≥65 years according to the Swedish guidelines for COPD [28], *AUC(CV*_*T*_*c*, area under the curve defined by the CV_T_ value (for details see ‘Methods’).

Technetium (^99m^Tc) was generated (Covidien, Dublin, Ireland) and thereafter Technegas was prepared and delivered from the Technegas generator according to the manufacturer’s instructions. Starting from functional residual capacity, subjects inhaled a deep breath of Technegas and hold their breath for 2 to 5 s and then expired. This manoeuvre was repeated until approximately 75 MBq ^99m^Tc (according to the gamma camera) had been deposited in the lungs. The subjects inhaled the gas in a supine position immediately before the ventilation SPECT acquisition in the same position. The data was acquired using a double-headed gamma camera (GE Infinia, Milwaukee, WI, USA) with a LEHR collimator. There were 120 projections (20 s each) equally spaced over 360°, and each projection was a 128 × 128 element matrix with a pixel size of 3.45 × 3.45 mm^2^. Auto-contouring and a 130 to 154 keV energy window were employed. The CV value of the two regions of interest (3 × 3 pixels) within the high-count area of the lung in two projections, based on the five healthy volunteers, was about 17%, consistent with the result of the simulated normal.

After the SPECT acquisition, without moving the patient, a low-dose CT examination was performed using the X-ray equipment mounted on the gamma camera (Hawkeye, GE Infinia, Milwaukee, WI, USA).

The effective dose for this protocol is estimated to be 3.1 mSv (1.1 mSv for SPECT [29] and 2 mSv for CT), which required a total acquisition time of 25 min.

### SPECT reconstruction and filtering

Each set of 20 noisy projections in the simulated COPD study and subject projections in the human study was reconstructed using the iterative ordered subset expectation maximisation reconstruction software developed at Johns Hopkins University, Baltimore, MD, USA. The reconstruction included correction for attenuation, scatter and collimator detector response (CDR). In the simulated COPD study, the phantom attenuation was corrected for using an attenuation map of the phantom, and in the human study the CT scans were used for attenuation correction. Scatter correction was performed using the effective source scatter estimation (ESSE) [23,30]. The ESSE model requires scatter kernel files, which were generated using Monte Carlo simulations with SIMIND. An analytic geometrical model for CDR compensation was used. Reconstructions were performed using ten iterations and 16 subsets. The side length of a voxel in a reconstructed image was for the simulated COPD study and human study with 3.30 mm and 3.45 mm respectively. The reconstructed images were post-filtered with a Butterworth filter [31] with a cut-off frequency of 0.5 cm^−1^ and a power of 6 (i.e. order 3).

### Method for analysis of inhomogeneities

**Figure 2:**
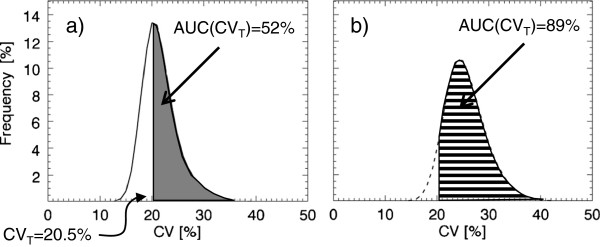
**Illustration of the area under the curve for CV-values greater than the threshold value CV**_**T**_**.** The mean frequency functions of 20 noise realisations of the CV values corresponding to **(a)** distribution 1, the simulated normal, and **(b)** distribution 8, ‘2-cm^50%^ 48% evenly.’ CV_T_ was set to 20.5% for the simulated distributions. Indicated in the figure are the calculated AUC(CV_T_) values for the two distributions.

The 3D reconstruction was subjected to a 3 × 3 × 3 cm^3^ (i.e. 9 × 9 × 9 voxels) cubic kernel that stepped through the volume. A step was a one-voxel-step in the *x*, *y* or *z* direction. In each step, CV was calculated. An additional file shows the behaviour of the CV value (see Additional file 1). The kernel defined which voxels of the reconstruction the CV calculations were based on. Voxels outside the defined lung volume were excluded from the CV analysis. The CV values were stored in the middle position of the kernel in a new matrix, the CV matrix, with the same matrix size as the reconstruction. The CV values for each reconstruction were also plotted as a frequency function with an area under the curve (AUC) of 100% (see Figure 2). The relative numbers of CV values in bins of width of 1% were used to construct the frequency functions.

#### Defining the extension of the lung

For the simulated COPD study, the lung voxels in distribution 1 (Table 1), containing values greater than half the maximum lung value, were set to be the lung.

For the human study, semi-automated lung segmentation was used on each subject based on the individual CT acquisition and an empirical linear attenuation coefficient threshold value of 0.12 cm^−1^. The linear attenuation coefficients for lung tissue and soft tissue are approximately 0.04 and 0.16 cm^−1^, respectively (ICRU 44). Therefore, the defined lung for each subject in the human study will include the whole lung cavity.

#### Edge layers

Due to the limited spatial resolution of the SPECT system, with a full width at half maximum (FWHM) of about 1 to 1.5 cm, the reconstructed activity distribution around the edge of the lung will be blurred. Voxels involved in the lung edge will result in high CV values, which are not necessarily correlated to inhomogeneity of the lung activity distribution. Therefore, a one-voxel-wide layer of the lung was ‘peeled off’ from the periphery of the segmented lung, thereby creating a reduced lung volume with most of this edge effect excluded.

#### Threshold values and area under curve

The area under the frequency function (AUC) for CV values greater than a threshold value for CV, called CV_T_, was defined as AUC(CV_T_) and expressed as the percentage of the total AUC (Figure 2). Since the future aim is to discriminate between healthy distributions and less severe COPD distributions, the optimal CV_T_ value was evaluated by finding the largest separation between corresponding AUC(CV_T_) values for the healthy ‘uniform’ and the ‘1-cm^50%^ 10% evenly’ distributions (distributions 1 and 2 in Table 1) in the simulated COPD study. CV_T_ values ranging from 1% to 25% were evaluated. The largest separation was found for the CV value corresponding to the peak value of the mean frequency function of the healthy distributions, i.e. the modal value, plus about 1%. The modal value was used for simplicity.

In the simulated COPD study, the healthy mean frequency function is based on the 20 noise realisations of the uniform activity distribution; and in the human study, it is based on the activity distributions of the five healthy volunteers. AUC(CV_T_) was then calculated for all activity distributions in both the simulated COPD study and the human study.

In the simulated COPD study, the group of 20 AUC(CV_T_) values, based on the 20 noise realisations of the simulated normal distribution, was compared with the corresponding groups of simulated COPD distributions, using the non-parametric Mann–Whitney *U* test and Statistica (version 9, StatSoft, Tulsa, Oklahoma, USA). This test was used because no assumption of normal distributions was made. The same test was performed comparing the five COPD patients with the five healthy volunteers.

### Image and computer processing

The addition of Poisson noise, post-filtration and evaluation were performed using in-house software developed in Interactive Data Language (IDL; ITT Visual Information Solutions, Boulder, CO, USA).

## Results and discussion

### Results of the Monte Carlo-simulated COPD study

**Figure 3:**
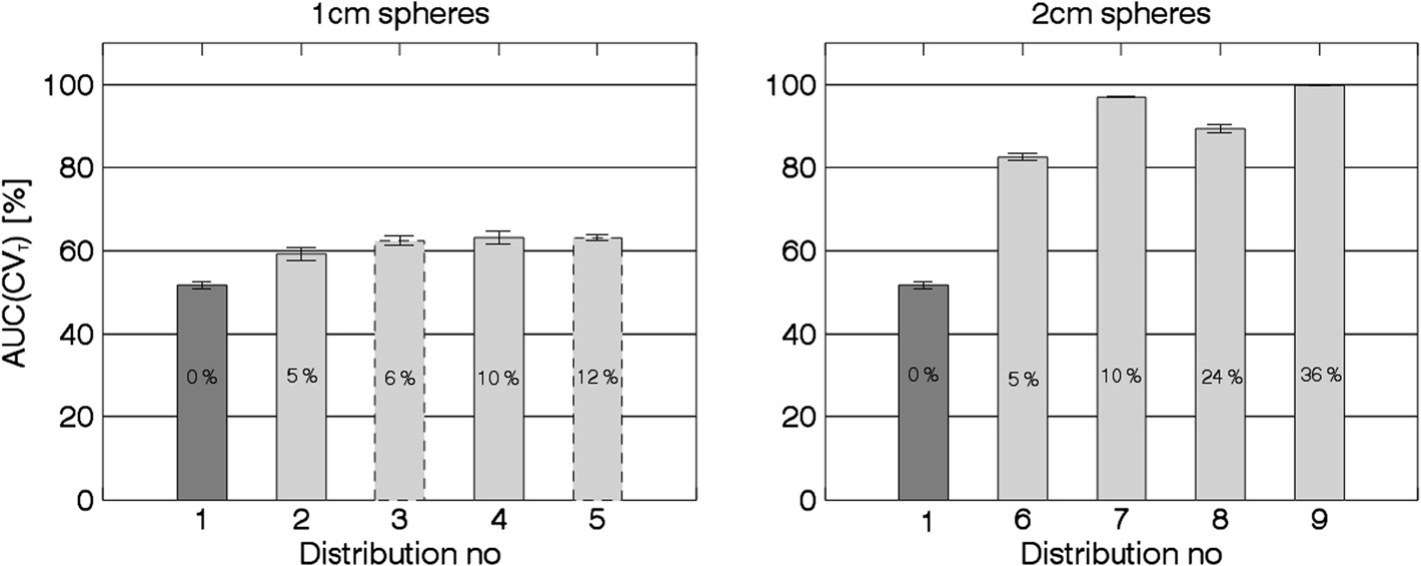
**The mean AUC(CV**_**T**_**) value for each activity distribution as listed in Table**1**.** The dark grey bars show simulated normal, and the light grey bars show simulated COPD. Bars with dashed outline correspond to clustered distributions. The total reduction of ventilation for each distribution is written in the bars. The error bars show a 95% CI, based on the 20 noise realisations.

The AUC(CV_T_) was calculated for all simulated activity distributions using a CV_T_ = 20.5%, as illustrated in Figure 2. Figure 3 shows that the AUC(CV_T_) values for all simulated COPD distributions tested [reflecting COPD changes of different severity (distributions 2 to 9)] can be separated statistically from the corresponding value of the simulated normal distribution of the healthy lung (distribution 1). The mean AUC(CV_T_) value for the simulated normal distribution is 52% ± 1% (95% confidence interval (CI)). For simulated COPD distributions with lesions with a diameter of 1 cm, corresponding values range from 58% to 65%, and for lesions with a diameter of 2 cm, from 82% to 100%. Comparing AUC(CV_T_) values for distributions with the same lesion size and localisation but different activity levels (i.e. comparing the distributions 2 with 4, 3 with 5, 6 with 7 and 8 with 9) shows that the one with the lowest activity level in the lesions has the highest AUC(CV_T_) value with one exception. For distributions 3 and 5, no difference in AUC(CV_T_) values are found. There is a statistically significant difference (*n*_1_ = *n*_2_ = 20, *p* < < 0.001, two-tailed) between the AUC(CV_T_) values for the 20 noise realisations of distribution 1 (the simulated normal) and distribution 2 (1 cm^50%^ 10% evenly). Comparing AUC(CV_T_) values for distributions with the same lesion size and activity levels but different localisation shows a difference between distributions 2 and 3 (with a total reduction of ventilation of 5% and 6% respectively, *p* < 0.002), but not between 4 and 5 (with a total reduction of ventilation of 10% and 12% respectively).

**Figure 4:**
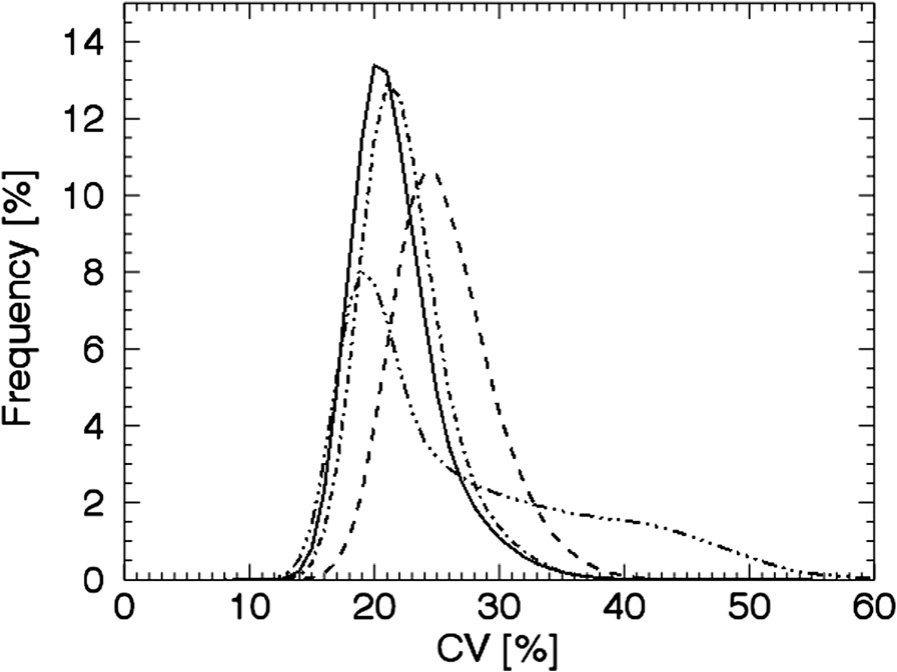
**Frequency functions of the CV- values for simulated distributions.** The mean frequency functions of 20 noise realisations of the CV values corresponding to distribution 1, the simulated normal (solid line); distribution 4, ‘1 cm^0%^ 10% evenly’ (dash-dotted); distribution 5, ‘1 cm^0%^ 12% centred’ (dash-dot-dot-dotted); and distribution 8, ‘2 cm^50%^ 48% evenly’ (dashed).Only four curves out of nine are shown for clarity.

Figure 4 shows that the differences shown in Figure 3 are due to shifts of the frequency functions, i.e. the proportion of high CV values is higher for the simulated COPD distributions compared to the simulated normal. The frequency function of the clustered distribution 5 (1 cm^0%^ 12% centred) has a larger proportion of high CV values compared to the ‘evenly’ distributions (see Figure 4). For the clustered distribution 3 (1 cm^50%^ 12% centred) this tale towards high CV values is less pronounced, and its frequency function is in between distribution 4 (1 cm^0%^ 10% evenly) and 5.

**Figure 5:**
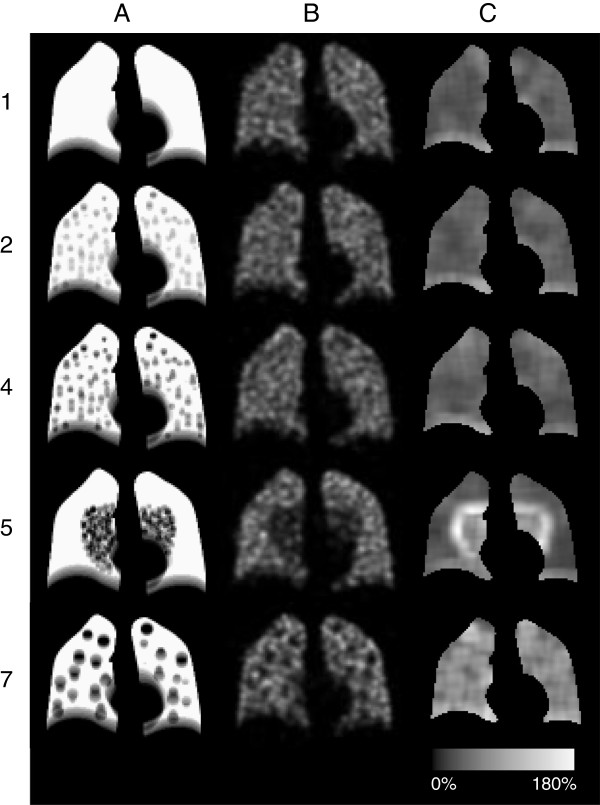
**Reconstructed images and CV matrices for simulated distributions.** Each row shows coronal slices based on the activity distributions 1, 2, 4, 5 and 7 in Table 1, **(A)**; the activity distribution, **(B)**; a filtered reconstruction and, **(C)**; corresponding CV matrix.

Figures 3 and 4 show that with the presented method we can identify a ventilation reduction not visible to the human eye in the reconstructed images, as shown in Figure 5 (row 2, column B). The CV values corresponding to evenly distributed 1 cm lesions are relatively constant (rows 2 and 3, column C) compared to the CV values corresponding to evenly distributed 2 cm lesions (distribution 7 row 5, column C) where the surfaces of the lesions are visible. The CV matrix for the clustered distribution clearly shows the surface of the cluster (distribution 5 row 4, column C). Due to the limited number of counts in the acquired projections there are fluctuations inside the lung even for the simulated normal. Notably, respiratory motions give rise to higher CV values at the base of the lung compared to the apex (column C), and, consequently, are not a result of reduced ventilation.

### Results of the human study

**Figure 6:**
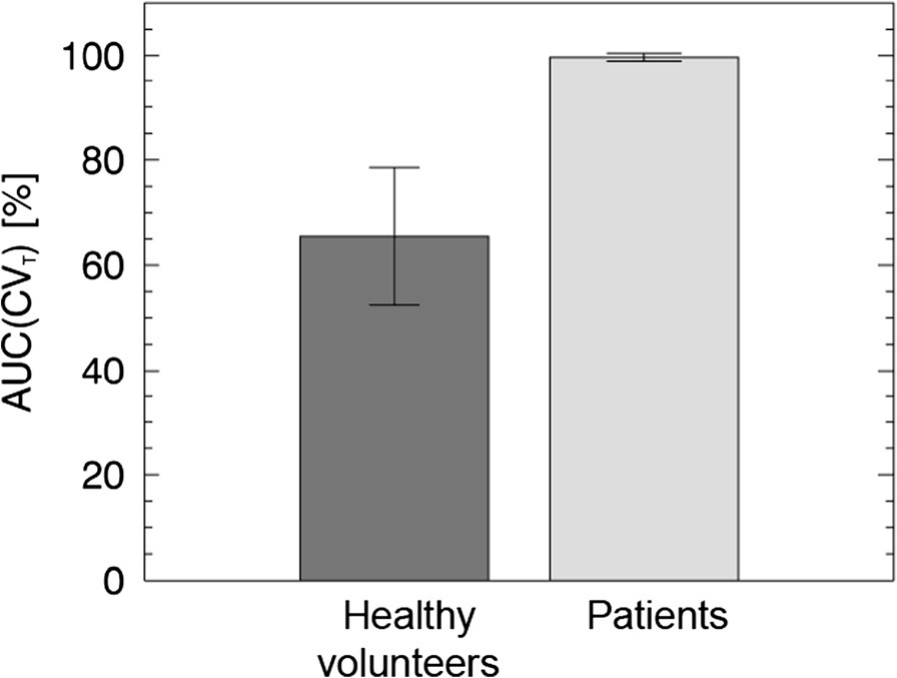
**The mean AUC(CV**_**T**_**) for the human *in vivo *study with error bars representing the 95% CI.**

Figure 6 shows that the mean AUC(CV_T_) value for the healthy volunteers was 65% ± 13% (95% CI), while the mean AUC(CV_T_) value for the patients was 100% ± 1% (95% CI). There was a statistical significant difference (*n*_1_ = *n*_2_ = 5, *p* <0.05, two-tailed) between the AUC(CV_T_) values for the volunteers and the patients.

**Figure 7:**
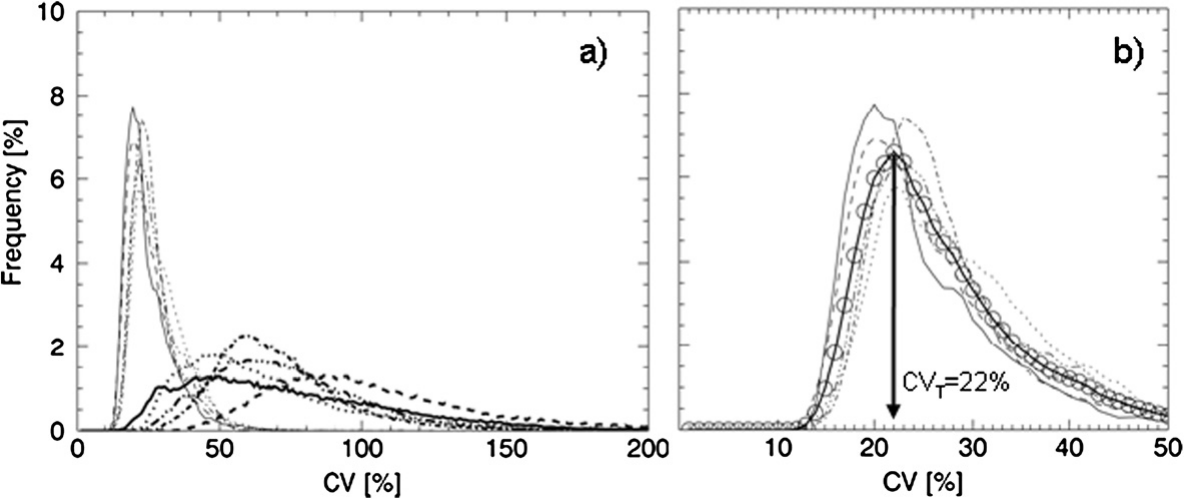
**Frequency functions of the CV values for the human study. (a)** The frequency functions of the CV values corresponding to healthy subject H1 (solid line), H2 (dotted), H3 (dashed), H4 (dash-dotted), H5 (dash-dot-dotted), and patients with bold line styles; P1 (solid line); P2 (dotted); P3 (dashed); P4 (dash-dotted); P5 (dash-dot-dotted). **(b)** An expanded view of the frequency functions for the healthy subjects and their mean frequency function (bold line with open circles). The arrow shows the CV threshold value for the human study at 22%.

The *in vivo* study also shows the expected substantial difference between the healthy volunteers and the patient group when all CV frequency functions are summarised, Figure 7a. The curves of the healthy volunteers are gathered to the left on the abscissa and are more peaked in shape compared to the corresponding curves of the patients, which are shifted more to the right on the abscissa and are wider in shape. In Figure 7b, the mean CV curve of all five healthy volunteers is shown, from which a CV threshold value, CV_T_ = 22%, is obtained. The calculated AUC(CV_T_) values of all human subjects are summarised in Table 2.

**Figure 8:**
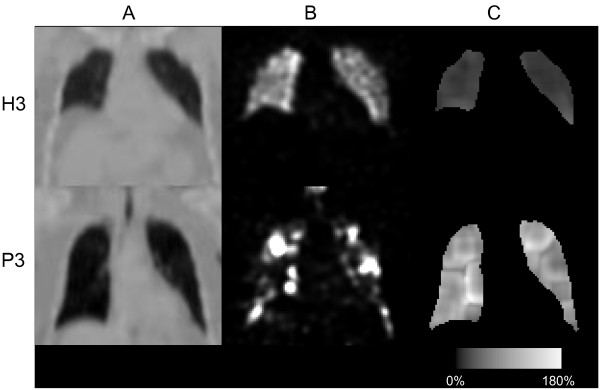
**Reconstructed images and CV matrices for healthy volunteer H3 and patient P3.** One row for healthy volunteer H3 and one row for patient P3, showing **(A)**; coronal slices of the attenuation map, **(B)**; a filtered reconstruction and, **(C)**; corresponding CV matrix.

Figure 8 shows reconstructed images and CV matrices for healthy volunteer H3 and patient P3. Notably, while the healthy volunteers all demonstrate high CV values in the lung bases due to respiratory motions, this was not observed in patients (column C), probably because of restricted movement of the diaphragm. Patients with advanced emphysema are expected to demonstrate hot spots in their ventilation distributions. Cubic-shaped areas with the size of the cubic kernel could be seen around the hot spots, and these contributed to the wide distribution of the frequency functions of the patients (see Figure 7a). However, also in volumes without hot spots, higher CV values were found in the patients than in the healthy volunteers.

## Discussion

In the present study, we have modified a method by Xu et al. in order to improve the quantification of ventilation inhomogeneities in a phantom model of a COPD lung. In contrast to what has been previously reported, our improved method was able to assess even minor COPD changes by using the AUC(CV_T_) value, as a global value of ventilation inhomogeneities, and to discriminate these changes from a model of a healthy homogeneous lung. The present pilot study also shows that our way of performing lung SPECT and calculating the AUC(CV_T_) significantly discriminates non-smoking healthy volunteers from patients with advanced COPD.

The NCAT software is able to create thorax voxel phantoms of a human, based on a fine-segmented male [32]. The NCAT software is flexible since different sizes and shapes of different tissues can be selected and natural movements caused by heartbeat and respiration can be modelled. Because most of the COPD patients are elderly [33], we decided to use a lung volume corresponding to a 65-year-old male. Low-ventilated regions, associated with anatomical changes of COPD, are distributed in patients in various ways, and these volumes can vary between 0.5 mm (the size of a few alveoli) and several centimetres in diameter. One of the aims of the present study was to mimic mild to moderate changes of COPD, and therefore small lesions were of interest. Since the spatial resolution of the SPECT system is about 1 to 1.5 cm (expressed in FWHM), the lesions modelled had a diameter of 1 and 2 cm. Lacking previous studies on the distribution of ventilation inhomogeneities in mild COPD, we assumed that COPD lesions are either evenly distributed in the whole lung volume or centred in clusters (see Table 1). In this way, our method was evaluated on two groups with completely different lesion distributions. The density of the lesions was approximated to be the same as for the healthy lung tissue since we aimed to model less severe changes of COPD. Indeed, activity distribution 1 cm^50%^ 10% (distribution 2 in Table 1) illustrates activity inhomogeneities that are almost too small for the SPECT system to resolve, and, clearly, not visible to the human eye in the reconstructed image (*cf*. Figure 5). Thus, we believe this distribution would be a good representative of a case of mild COPD. However, it should be pointed out that the activity distributions selected in the different cases were not primarily chosen because of their consistency with biology, but more because of our ability to unambiguously describe them.

*How does the magnitude of the present volume of ventilation defects compare to reductions of spirometric variables*? Fifty percent reduction of the ventilation in 10% of the lung volume (distributions 2 and 6 in Table 1) corresponds to the 5% total reduction of the functioning lung tissue. Although not exactly comparable, reductions of spirometric variables of similar magnitudes are likely to remain undetected, as the normal range of spirometric variables is roughly ±15% to 20%. Thus, in our anthropomorphic phantom, we consider most of the present volume of ventilation defects as comparatively small.

When considering the result of the presented quantitative method, a number of important parameters have to be accounted for, e.g. count density (statistical noise), collimator, number of iterations and subsets, reconstruction compensations and post-filtering. In this work we used clinically relevant values for these parameters. Other important factors are the method-specific ones, i.e. the lung edge effects and kernel size that we evaluated. The resolution of the SPECT system is limited, and therefore, high CV values will always be found in the periphery of the healthy lung. Lung edges with low activity lesions will instead give lower CV values. High CV values due to healthy edges reduce the differences between frequency functions from healthy and unhealthy activity distributions; i.e. they also reduce the separation between corresponding AUC(CV_T_) values. Therefore, the CV analysis was performed in a volume that had part of the edge effect excluded. Exclusion of a one-voxel layer, however, removes 21% of the phantom lung and 20% to 29% of the human lung parenchyma from the analysis, which is why small lesions in the periphery might not be detected. The kernel approach used does not exclude any additional volume of the lung in the analysis. Kernels with five different side lengths were evaluated in the simulated COPD study, i.e. 1.0, 1.7, 2.3, 3.0, 3.6 and 4.3 cm. Increasing the side length resulted in an increasing differentiation between the AUC(CV_T_) values of the simulated normal and 1 cm^50%^ 10% evenly distribution (distributions 1 and 2 in Table 1). However, for the two largest cube sizes tested, in combination with some values of the above-mentioned parameters (e.g. LEHR collimator, a Butterworth post-filter with a cut-off frequency of 0.3 cm^−1^ and a power of 6, 125 MBq, ten iterations and 16 subsets and no exclusion of edge voxels), the CV frequency functions of the simulated normal distribution were double peaked. Therefore, to minimise the risk of double peaks in a clinical setting, we chose a kernel with the side length of 3 cm, including 729 voxels.

The present method of quantitative analysis has two major advantages. Firstly, it discriminates cases with the same loss of ventilation, but with inhomogeneities differently distributed and with different lesion sizes, from the simulated normal lung, even when COPD changes are minor. For example, distributions 2 (1 cm^50%^ 10% evenly), 3 (1 cm^50%^ 12% centred) and 6 (2 cm^50%^ 10% evenly) in Figure 3 all represent only a 5% to 6% total reduction of ventilation, but their resulting AUC(CV_T_) values are well above the value of the normal lung. The same holds true for distributions 4 (1 cm^0%^ 10% evenly), 5 (1 cm^0%^ 12% centred) and 7 (2 cm^0%^ 10% evenly), which all correspond to a 10% to 12% total reduction of ventilation. Secondly, for the same lesion size, increasing AUC(CV_T_) values tend to correlate with decreasing total ventilation. For example, distribution 6 (2 cm^50%^ 10% evenly) in Figure 3, corresponding to a 5% total reduction of ventilation, gives a lower AUC(CV_T_) value than distribution 7 (2 cm^0%^ 10% evenly) corresponding to a 10% total reduction of ventilation. But clearly, even with the same loss of ventilation, activity distributions with a few large lesions (≥1.5 cm) with low activity will give higher AUC(CV_T_) values than activity distributions with many small lesions (≤1.5 cm) with relatively high activity in each lesion (e.g. see Figure 3 and Figure 5, distributions 4 and 7 at rows 3 and 5, column C).

It is desirable that distributions with the same total reduction of ventilation result in the same level of AUC(CV_T_) values independent of lesion shape and distribution. This is the case of the evenly distribution 4 and the clustered distribution 5 (with a total reduction of ventilation of 10% to 12%). However, the difference in distribution can be seen in the frequency functions (Figure 4) and in corresponding CV matrices (Figure 5). Clusters positioned in other parts of the lung have not been investigated. Factors influencing the result are the volume and activity concentration of the spheres in the cluster and the size of the cluster’s surface area towards the uniform part of the lung, and not where in the lung the cluster is positioned.

One limitation of the present method is its loss of sensitivity when COPD changes become more advanced. An activity distribution with an AUC(CV_T_) value close to 100% can easily be separated from the simulated normal distribution; however, further reduction of ventilation will only result in an almost unchanged AUC(CV_T_) value (because 100% is the highest possible value). On the other hand, the present method is customised to detect early and minor COPD changes and not to be a diagnostic for advanced emphysema, for which methods such as HRCT are more useful. Furthermore, in cases of advanced COPD, the appearance of the frequency function of CV values can be used directly, without calculating the CV_T_ and the AUC(CV_T_), to estimate the COPD severity.

The activity distribution in healthy humans is not as homogeneous as the simulated normal distribution used in the simulated COPD study, which might be due to the shape of the bronchial tree and the gravity influencing the lung. This difference is seen in the defined CV_T_ values, 20.5% for the simulated COPD study and 22.0% for the human study. Furthermore, the human thorax exhibits a large variety of sizes and shapes. In order to decrease the influence of the lung size, AUC(CV_T_) values are presented in a percentage of total lung volume instead of absolute values. This prevents large healthy lungs giving high AUC(CV_T_) values and small inhomogeneous lungs giving low AUC(CV_T_) values. However, a larger 95% CI of the AUC(CV_T_) value of the healthy volunteers of ±13% around the mean compared to the simulated COPD study of ±1% was found. This large variation of the healthy volunteers might be due to genetic variations, age effects, different histories of occupational and environmental exposures of noxious particles and gases e.g. passive exposure to tobacco smoke and varying techniques of inhaling the Technegas. The total amount of inhaled Technegas, expressed in megabecquerel, also affects the resulting CV values. A reconstructed ventilation distribution based on a low activity level will contain higher statistical noise compared to a distribution based on a higher activity level. Higher noise levels will result in higher CV values. A variation in the amount of inhaled Technegas was observed between the human subjects. Therefore, due to different activity levels, small shifts along the CV axis of the frequency functions in Figure 7 are present. Procedures resulting in more reproducible amounts of inhaled Technegas and normalisation methods that after image acquisition can compensate for such shifts will be further investigated. Reproducibility may be improved also by control of the depth and inhalation flow rate of the Technegas administration. In particular, the inhalation flow rate influences the ventilation distribution in normal subjects [34] and almost certainly also the deposition in patients with COPD.

The activity distribution in the COPD patients was characterised by areas of low activity and areas of high activity i.e. hot spots. Low activity areas are caused by reduced regional ventilation resulting in low Technegas particle deposition. Reduced regional ventilation may be caused by local obstruction of peripheral airways and/or by emphysematous areas with low elasticity. Hot spots on the other hand, as pointed out by Pellegrino et al. [35], may be caused by obstruction of central airways probably resulting in some Technegas impaction during inspiration but in particular by facilitating regional airflow limitation during expiration. Airflow limitation implies pronounced and oscillating narrowing of airway walls causing strong turbulence resulting in high impaction of Technegas particles during expiration [35]. These hot spots were included in the assessment of the CV values. This might be considered as a weakness of the present method, since the intention behind the use of CV values was to identify low activity volumes as an indication of disease and not to generate high CV values due to abnormal uptake. On the other hand, such hot spots are typical of the COPD lung and might therefore be accepted as contributing to the analysis. How these hot spots influence the analysis will be evaluated in our future work. Presently, we investigate if the present method is reliable enough for detection of COPD changes in smokers without manifested COPD.

## Conclusion

The proposed method generates as a global measure of ventilation inhomogeneities, an AUC (CV_T_) value that can differentiate with statistical significance between the activity distributions corresponding to varying severity of COPD changes and the healthy normal activity distribution in a breathing lung phantom. The method can also clearly differentiate between five patients with COPD of grades 3 and 4, and five healthy non-smokers. Therefore, the AUC (CV_T_) method is promising for assessment of COPD inhomogeneities in the lung SPECT ventilation image. The potential of the AUC(CVT) method to detect less advanced COPD in patients, e.g. smokers without manifested COPD, needs further investigations.

## Abbreviations

AUC: the area under the curve; AUC(CVT): the area under the curve for CV values greater than CV_T_; CDR: collimator detector response; COPD: chronic obstructive pulmonary disease; CV: the coefficient of variance; CVT: CV threshold value; DLCO: diffusion capacity of the lung for carbon monoxide; ESSE: effective source scatter estimation; FEV1: forced expiratory volume in one second; FWHM: full width at half maximum; HRCT: high-resolution computed tomography; LEHR: low-energy high-resolution; RV: residual volume; SPECT: single-photon emission computed tomography; TLC: total lung capacity; VC: vital capacity

## Competing interests

The authors declare that they have no conflict of interest.

## Authors' contributions

PN participated in study planning, selected healthy controls, performed all lung SPECT analysis, evaluated data and wrote the manuscript. HLP participated in planning of the human study, selected patients, evaluated the data and wrote the manuscript. GAC and BB participated in planning and writing. MK participated in writing. MS and AG planned the study, evaluated data, oversaw the writing, and provided grant funding. All authors read and approved the final manuscript.

## Supplementary Material

Additional file 1: Figure S1. A slice through a 3D geometry with a background mean level of 100 and spheres at 55 and 5 with Poisson noise incorporated, and an outlined kernel, are shown at the top. The CV, SD and mean values for the central position of the kernel are plotted in the diagram. Measurements above a) correspond to the large sphere with a mean level of 55 at the top to the left, b) to the small 55-level sphere, second to the right, c) to the large 5-level sphere, third to the right, and d) to the small 5-level sphere at the far right. Click here for file
