# Supplementary material for: Quantitative lung SPECT applied on simulated early COPD and humans with advanced COPD
Source: EJNMMI Res. 2013 Apr 19;3:28. doi: 10.1186/2191-219X-3-28 (PMC3706344; doi:10.1186/2191-219X-3-28)

The behaviour of the CV-value

To illustrate the behaviour of the CV-value (Equation 1) a cubic kernel stepping through a simplified 3D geometry with two different sphere sizes and two different count levels are shown in Figure S1.

$$CV = \frac{SD}{mean} \cdot 100 \, [\%] \qquad (1)$$

CV will be highest when the kernel is positioned over a large sphere containing a low count level (c in Figure S1). A large sphere with intermediate count level (a in Figure S1) will result in a somewhat higher CV-level than a small sphere with a low count level (d in Figure S1). A small sphere with intermediate count level (b in Figure S1) will have a CV-level just a little higher than the background.

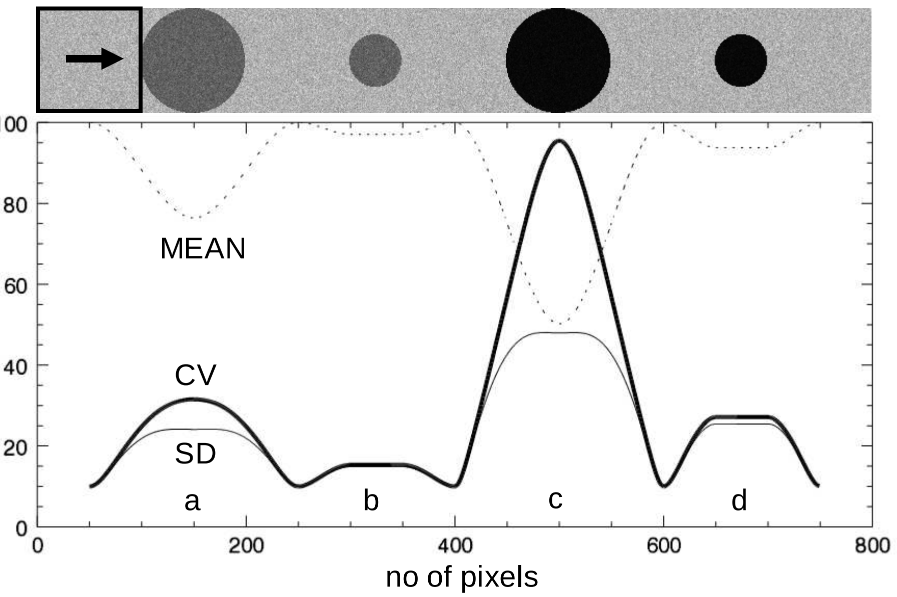

Supplement: Additional file 1: Figure S1. — A slice through a 3D geometry with a background mean level of 100 and spheres at 55 and 5 with Poisson noise incorporated, and an outlined kernel, are shown at the top. The CV, SD and mean values for the central position of the kernel are plotted in the diagram. Measurements above a) correspond to the large sphere with a mean level of 55 at the top to the left, b) to the small 55-level sphere, second to the right, c) to the large 5-level sphere, third to the right, and d) to the small 5-level sphere at the far right. [file 2191-219X-3-28-S1.pdf]
